# RpoN Regulates Virulence Factors of *Pseudomonas aeruginosa* via Modulating the PqsR Quorum Sensing Regulator

**DOI:** 10.3390/ijms161226103

**Published:** 2015-11-30

**Authors:** Zhao Cai, Yang Liu, Yicai Chen, Joey Kuok Hoong Yam, Su Chuen Chew, Song Lin Chua, Ke Wang, Michael Givskov, Liang Yang

**Affiliations:** 1 Singapore Centre for Environmental Life Sciences Engineering (SCELSE), Nanyang Technological University, Singapore 637551; caiz0013@e.ntu.edu.sg (Z.C.); liu_yang@ntu.edu.sg (Y.L.); chenyc@ntu.edu.sg (Y.C.); yamk0002@e.ntu.edu.sg (J.K.H.Y.); schew005@e.ntu.edu.sg (S.C.C.); chuasonglin@nus.edu.sg (S.L.C.); mgivskov@sund.ku.dk (M.G.); 2 Interdisciplinary Graduate School, Nanyang Technological University, Singapore 637551; 3 Department of Respiratory Disease, First Affiliated Hospital of Guangxi Medical University, Nanning 530000, China; keewang@hotmail.com; 4 Costerton Biofilm Center, Department of International Health, Immunology and Microbiology, University of Copenhagen, 2200 København N, Denmark; 5 School of Biological Sciences, Nanyang Technological University, Singapore 637551

**Keywords:** *Pseudomonas aeruginosa*, *rpoN*, *pqsR*, quorum sensing

## Abstract

The alternative sigma factor RpoN regulates many cell functions, such as motility, quorum sensing, and virulence in the opportunistic pathogen *Pseudomonas aeruginosa* (*P. aeruginosa*). *P. aeruginosa* often evolves *rpoN*-negative variants during the chronic infection in cystic fibrosis patients. It is unclear how RpoN interacts with other regulatory mechanisms to control virulence of *P. aeruginosa*. In this study, we show that RpoN modulates the function of PqsR, a quorum sensing receptor regulating production of virulence factors including the phenazine pyocyanin. The ∆*rpoN* mutant is able to synthesize 4-quinolone signal molecule HHQ but unable to activate PqsR and *Pseudomonas* quinolone signal (*pqs*) quorum sensing. The ∆*rpoN* mutant produces minimal level of pyocyanin and is unable to produce the anti-staphylococcal agents. Providing *pqsR* *in trans* in the ∆*rpoN* mutant restores its *pqs* quorum sensing and virulence factor production to the wild-type level. Our study provides evidence that RpoN has a regulatory effect on *P. aeruginosa* virulence through modulating the function of the PqsR quorum sensing regulator.

## 1. Introduction

Bacterial chronic infections raise a huge burden for public health today, which significantly prolong hospitalization period and increase treatment costs. It is well known that bacteria are able to adapt their genome and physiology during chronic infections [1,2,3]. For example, the opportunistic pathogen *Pseudomonas aeruginosa* (*P. aeruginosa*) is able to colonize in the airway of cystic fibrosis (CF) patient for decades [2]. Colonization in CF patients has a high frequency to select for mutations in *lasR*, *pvdS*, *mucA*, and *rpoN* genes of the *P. aeruginosa* genome [4,5]. Understanding how these genetic adaptations affect the bacterial physiology and the microbial ecology is essential for development of strategies for infection control.

One major feature of *P. aeruginosa* CF adaptation is the reduction of virulence. *P. aeruginosa* employs the cell-to-cell communication (quorum sensing) to regulate expression of a large set of virulence genes such as genes required for the synthesis of pyocyanin, elastase, proteases and iron siderophore pyoverdine [6,7]. Mutations in *lasR* and *pvdS* of CF isolates abolish the *las* quorum sensing and siderophore synthesis, respectively, and thus reduce *P. aeruginosa* virulence [4,5]. Mutations in *mucA* and *rpoN* genes of CF isolates are believed to be more important for the adaptive response of *P. aeruginosa* towards the host immune systems. The *mucA* mutation of CF *P. aeruginosa* isolates leads to conversion from non-mucoid to mucoid phenotype, characterized by an over production of the alginate polysaccharide [8]. Large amounts of alginate produced by the *mucA* mutants provide protection to the bacterial cells against the phagocytic cells [9]. The *rpoN* mutation of CF *P. aeruginosa* isolates leads to deficiency in surface pilus, flagellum synthesis and their mediated motilities [10], which confers the immune evasion capacity of the *P. aeruginosa* [11,12].

The *rpoN* mutation has a profound impact on *P. aeruginosa* by affecting metabolism, motility, biofilm formation and quorum sensing [4,13]. It is unclear how RpoN regulates quorum sensing genes in *P. aeruginosa* and whether this is going to affect the microbial ecology of CF lungs. Here, we showed that RpoN modulates the functions of the quorum sensing receptor PqsR, which determines the *Pseudomonas* quinolone signal (*pqs*) quorum sensing-regulated virulence factors and biofilm formation.

## 2. Results

### 2.1. RpoN Regulates P. aeruginosa pqs Quorum Sensing via PqsR

**Figure 1:**
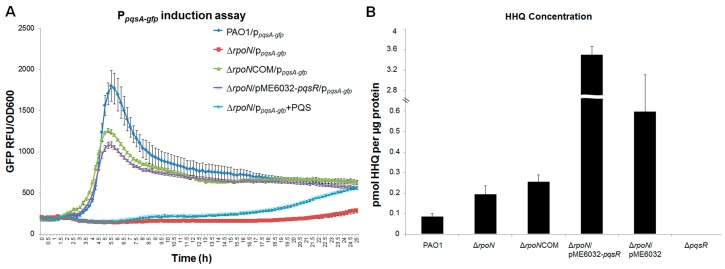
Regulation of *pqs* quorum sensing by RpoN. (**A**) Induction of p*_pqsA-_gfp* transcriptional fusion in PAO1 wild-type, Δ*rpoN*, Δ*rpoN*COM, Δ*rpoN*/pME6032*-pqsR* and Δ*rpoN* + PQS (2-heptyl-3-hydroxy-4(1H)-quinolone). Cultures were monitored for their gfp fluorescent protein (GFP) fluorescence by using a Magellen Tecan^®^ Infinite 200 PRO microplate reader. Means and standard deviations (S.D.) in relative fluorescence units (RFU) from triplicate experiments are shown; (**B**) High-performance liquid chromatography (HPLC) analysis of HHQ (4-hydroxy-2-heptylquinoline) production by PAO1, Δ*rpoN*, Δ*rpoN*COM, Δ*rpoN*/pME6032*-pqsR*, Δ*rpoN*/pME6032 and Δ*pqsR.* Means and S.D. from triplicate experiments are shown.

The ∆*rpoN* mutant is well known to be deficient in pyocyanin production, which is under direct control by the *Pseudomonas* quinolone signal (*pqs*)-mediated quorum sensing mechanism [14]. In the *pqs* quorum sensing system, auto-induction of the *pqsABCDE* operon is driven by the PqsR, which is known to bind to the *pqsA* promoter and induce its transcription in the presence of the 2-heptyl-3-hydroxy-4(1H)-quinolone (PQS) or 4-hydroxy-2-heptylquinoline (HHQ) [14]. To elucidate the regulatory role of RpoN on the *pqs* quorum sensing mechanism, we monitored the expression of the *pqsA* promoter-gfp fusion p*_pqsA_*-*gfp* in wild-type PAO1, ∆*rpoN* mutant and the ∆*rpoNCOM* complementary strain*.* We observed that the expression level of the p*_pqsA_*-*gfp* fusion in the ∆*rpoN* mutant is significantly lower compared to that in the wild-type PAO1 and the ∆*rpoNCOM* complementary strain (Figure 1A). HPLC analysis showed that the ∆*rpoN* mutant produced similar level of HHQ compared to the PAO1 (Figure 1B), confirming that the *pqsABCDE* operon is functional in the ∆*rpoN* mutant.

Furthermore, we found that addition of synthesized PQS to the ∆*rpoN* mutant was unable to affect the expression of the p*_pqsA_*-*gfp* fusion in the ∆*rpoN* mutant (Figure 1A), which indicates that there might be no functional PqsR in the ∆*rpoN* mutant. We thus evaluated the effect of over-expressing *pqsR* on the *pqs* signaling of the ∆*rpoN* mutant. Overexpressing *pqsR* under the *lac* promoter in a pME6032-*pqsR* vector in the ∆*rpoN* mutant restored its *pqs* signaling (Figure 1A). We also investigated the regulation of RpoN on *pqs* signaling using *P. aeruginosa* strains from another background mPAO1 and obtained similar results (Figure S1).

### 2.2. RpoN Regulates Virulence Factors and Interspecies Competition through pqs Signaling

**Figure 2:**
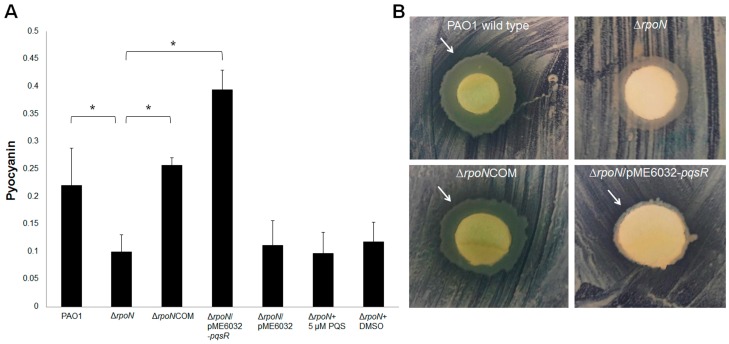
(**A**) Pyocyanin produced by PAO1 wild-type, ∆*rpoN*, Δ*rpoN*COM, ∆*rpoN*/pME6032-*pqsR* and Δ*rpoN*/pME6032 was determined by the chloroform extraction method. Means and S.D. from triplicate experiments are shown. Pyocyanin absorbance at OD_520 nm_ was normalized by culture cell density OD_600 nm_. Student’s *t*-test was performed for testing differences between groups. * *p* ≤ 0.05; (**B**) Inhibition of the growth of *Staphylococcus aureus* 15981 by (i) PAO1; (ii) ∆*rpoN*; (iii) Δ*rpoN*COM; and (iv) Δ*rpoN/*pME6032*-pqsR* on LB agar plates. White arrows indicate the inhibitory zones of growth.

The *pqs* quorum sensing regulates expression of virulence genes (e.g., pyocyanin biosynthesis genes) and mediates interspecies interactions and biofilm formation [15,16,17,18]. We then further examined whether RpoN affects these phenotypes in a *pqs*-dependent manner. Pyocyanin quantification assay showed that the ∆*rpoN* mutant produced much less pyocyanin compared to the wild-type PAO1 (Figure 2A). The deficiency in pyocyanin production of the ∆*rpoN* mutant was restored by both *rpoN* complementation and *pqsR* overexpression (Figure 2A). The control vector pME6032 has negligible effect on the pyocyanin production of the ∆*rpoN* mutant (Figure 2A).

**Figure 3:**
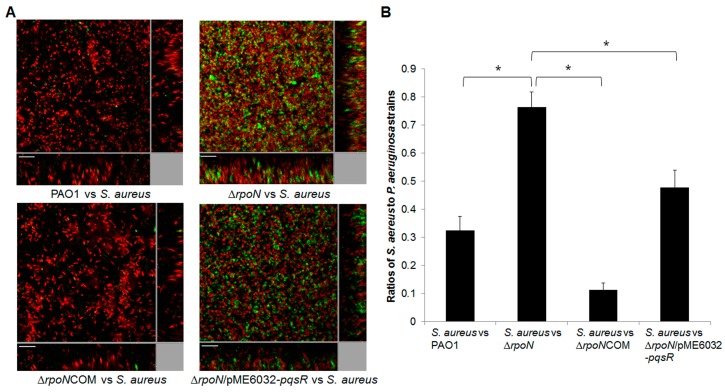
(**A**) Images of biofilm co-cultures of *S. aureus* 15981/pSB2019 with (i) PAO1; (ii) ∆*rpoN*; (iii) Δ*rpoN*COM and (iv) Δ*rpoN*/pME6032*-pqsR*, respectively. *S. aureus* 15981/pSB2019 appeared green due to GFP expression whereas *P. aeruginosa* strains were stained with red fluorescent dye CYTO62 used to generate the simulated 3D images (Bitplane, AG). Scale bar, 20 μm; (**B**) Biomass ratios of *S. aureus* to *P. aeruginosa* strains from different biofilm co-cultures were calculated using Imaris and shown in the histogram. Means and S.D. from triplicate experiments are shown. Student’s *t*-test was performed for testing differences between groups. * *p* ≤ 0.05.

Interspecies interactions play an important role during the progression of diseases, as most of the infections are polymicroibal in nature. *P. aeruginosa* coexists with many other microbial species during CF infections. One of the other dominant species in the CF airway is *Staphylococcus aureus* (*S. aureus*). *P. aeruginosa* was shown to inhibit *Staphylococcus* growth via the *pqs* quorum sensing-dependent mechanism [19,20]. We examined the impact of *rpoN* mutation on interactions between *P. aeruginosa* and *S. aureus*. We found that unlike the wild-type PAO1, the ∆*rpoN* mutant could not inhibit the growth of *S. aureus* in the plate growth assay (Figure 2B). The ∆*rpoN*COM complementation strain and the *pqsR* overexpressing ∆*rpoN/*pME6032-*pqsR* strain restored the capacity of the ∆*rpoN* mutant to inhibit the growth of *S. aureus* on LB agar plates (Figure 2B). We also examined the impact of *rpoN* mutation on interactions between *P. aeruginosa* and *S. aureus* in biofilm co-cultures. Similarly, we found that the ∆*rpoN* mutant gained less fitness against *S. aureus* in biofilm co-cultures compared to the PAO1 strain (Figure 3A,B). The ∆*rpoN*COM complementation strain had similar fitness to the PAO1 wild-type against the *S. aureus* in biofilm co-cultures. However, *pqsR* overexpression in the ∆*rpoN* mutant only partially restored its fitness against *S. aureus* in biofilm co-cultures (Figure 3A,B). This suggests that other factors regulated by RpoN but not by PqsR might play a role in competition between *P. aeruginosa* and *S. aureus* in biofilm co-cultures.

### 2.3. RpoN Mediates Killing of Caenorhabditis elegans through pqs Quorum Sensing

**Figure 4:**
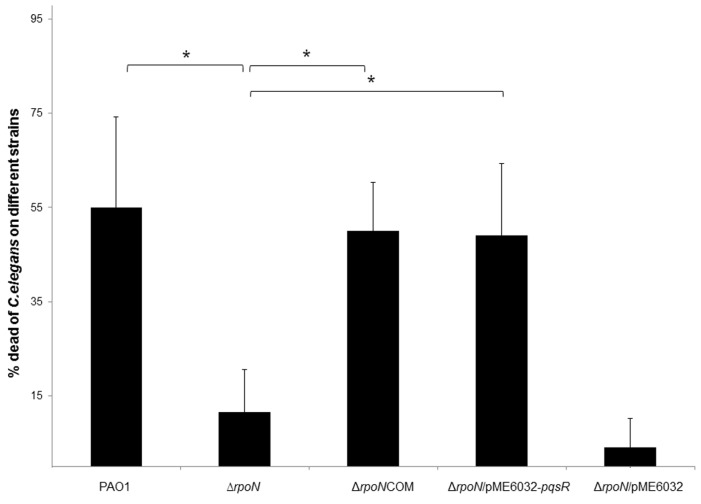
Death rates of *Caenorhabditis elegans* (*C. elegans*) growing on the lawn of different *P. aeruginosa* strains on agar plates. Means and S.D. from six replicates are shown. One-way ANOVA was performed for testing differences between groups. * *p* ≤ 0.05.

*P. aeruginosa* is able to kill *Caenorhabditis elegans* (*C. elegans*) using RpoN-regulated virulence products [21], we further examined whether *pqs* quorum sensing is involved in the RpoN-mediated killing of *C. elegans* by *P. aeruginosa*. As we expected, the death rate of *C. elegans* was much lower in the Δ*rpoN* mutant compared to the wild-type PAO1 strain (Figure 4). Δ*rpoN* mutants complemented with plasmids carrying either *rpoN* gene or *pqsR* gene restored its virulence against *C. elegans* (Figure 4). The death rate of *C. elegans* caused by ∆*rpoN*COM and Δ*rpoN*/pME6032-*pqsR* strains was similar but slightly lower than that of the wild-type PAO1 strain. The Δ*rpoN* mutant carrying pME6032 control vector expressed basal level of virulence only. These results are in accordance with the results we observed from pyocyanin quantification and p*_pqsA-_gfp* induction assay, suggesting that RpoN regulates virulence through PqsR.

### 2.4. Discussion

RpoN (σ54) is a conserved regulator in the bacterial kingdom that plays essential roles in regulating metabolism, motility and virulence of different species [22,23]. ∆*rpoN* mutants are selected during chronic adaptation of *P. aeruginosa* in the CF airways [4]. One of the reasons for this evolutionary trait is that the ∆*rpoN* mutant is able to escape the phagocytosis because of its deficiency in motility [10,12]. Another reason that *rpoN* mutation might be selected is due to the fact that the ∆*rpoN* mutant downregulates its virulence, which is also an important adaptation strategy for chronic CF infections [24,25]. It is unclear how RpoN regulates virulence in *P. aeruginosa*.

In the present study, we demonstrated that RpoN is able to regulate virulence factors via modulating the *pqs* quorum sensing. Specifically, our results suggest that PqsR is controlled by RpoN, which is in accordance with a recent study showing that RpoN binds with the *pqsR* sequence via ChIP-seq analysis [26].

Recent evidence suggested that nutrient clues could modulate *pqs* quorum sensing post-transcriptionally through the PqsR. For example, under oxygen limiting condition, the transcriptional regulator Anr is able to activate expression of the small non-coding RNA PhrS, which further stimulates translation of *pqsR* and activate *pqs* quorum sensing [27]. The small non-coding RNA CrcZ, which is required for sequester of the RNA-binding catabolite repression control protein Crc and Hfq in *Pseudomonas*, was also shown by us and others to negatively control *pqs* quorum sensing [18]. Hfq was shown to be able to bind to and stabilize the small non-coding RNA RsmY, which leads to abrogate of the RsmA, a global RNA-binding posttranscriptional regulator that can repress quorum sensing in *P. aeruginosa* [28]. Further studies should be carried out to investigate the roles of PhrS and CrcZ in mediating the regulation of RpoN on *pqs* quorum sensing in *P. aeruginosa* as well as in other species.

## 3. Experimental Section

### 3.1. Bacterial Strains, Plasmids, and Growth Conditions 

Bacterial strains and plasmid vectors used in this study are listed in Table 1.

**Table 1:** Bacterial strains, plasmids and primers used in this study.

Strain(s) or Plasmid	Relevant Characteristic(s)	Source or Reference
*P. aeruginosa* strains		
PAO1	Prototypic wild-type strain	[13]
Δ*rpoN*	Gm^r^; *rpoN* derivative of PAO1 constructed by allelic exchange	[13]
Δ*rpoN*COM	Gm^r^;Tc^r^; Δ*rpoN* carrying the pME6031-*rpoN* vector	This work
Δ*rpoN/*pME6032*-pqsR*	Gm^r^;Tc^r^; Δ*rpoN* carrying the pME6032-*pqsR* vector	This work
Δ*rpoN/*pME6032*-pqsR/p_pqsA-_gfp*	Gm^r^;Tc^r^; Carb^r^; Δ*rpoN/*pME6032*-pqsR* carrying the p*_pqsA-_gfp* vector	This work
Δ*rpoN*COM*/p_pqsA-_gfp*	Gm^r^;Tc^r^; Carb^r^; Δ*rpoN*COM carrying the p*_pqsA-_gfp* vector	This work
Δ*rpoN/*pME6032	Gm^r^;Tc^r^; Δ*rpoN* carrying the pME6032 vector	This work
Δ*pqsR*	*pqsR* derivative of PAO1 constructed by allelic exchange	[15]
*Staphylococcus aureus*		
15981	Prototypic wild-type strain	[29]
15981/pSB2019	Chl^r^; 15981 carrying the pSB2019 gfp-expressing vector	[29]
Plasmids		
pME6031	Tc^r^; Broad-host-range cloning vector	[30]
pME6031-*rpoN*	Tc^r^; pME6031 carrying the *rpoN* gene	[4]
pME6032	Tc^r^; broad host range vector	[30]
pME6032-*pqsR*	Tc^r^; pME6032 carrying the *pqsR* gene	[15]
p*_pqsA-_gfp*	Gm^r^;Carb^r^; pUCP22 carrying the *pqsA-gfp* transcriptional fusion	[16]

The *Escherichia coli* (*E. coli*) DH5a lab strain was used for standard DNA manipulations and plasmid maintenance. LB medium [31] was used for cultivation of *E. coli* strains. *P. aeruginosa* strains were cultivated in ABT minimal medium [32] supplemented with 2 g glucose·L^−1^ and 2 g casamino acids·L^−1^ (ABTGC) at 37 °C. King’s medium A (Sigma-Aldrich, Singapore) was used for the *P. aeruginosa* cultivation for the pyocyanin assay. Batch cultivation of *S. aureus* was carried out at 37 °C in Tryptic Soy Broth (TSB) medium (BD Biosciences, Singapore). The LB medium was supplemented with 100 µg ampicillin (Ap)·mL^−1^, 15 µg gentamicin (Gm)·mL^−1^, 15 µg tetracycline (Tc)·mL^−1^, 8 µg chloramphenicol (Cm)·mL^−1^ for plasmid maintenance in *E. coli* when necessary. The TSB medium was supplemented with 10 µg chloramphenicol (Cm)·mL^−1^ for plasmid maintenance in *S. aureus*. The ABTGC medium was supplemented with 30 µg Gm·mL^−1^, 50 µg Tc·mL^−1^, 200 µg carbenicillin (Cb)·mL^−1^ for marker selection in *P. aeruginosa* when necessary.

### 3.2. HHQ Quantification by High Performance Liquid Chromatography (HPLC)

*P. aeruginosa* strains were grown in triplicates in 25 mL of ABTGC medium at 37 °C, 200 rpm for 8 h until entering early stationary phase. Cultures were centrifuged (10,000× *g*, 10 min) and 20 mL of supernatants were filtered through the 0.22 µm Hydrophilic Cartridge Filters (Millipore, Singapore). HHQ was extracted by 10 mL of acidified ethyl acetate for three times. The ethyl actate fraction was dried and the residue was re-suspended in 200 µL of isopropal alcohol as previously described [33]. The concentration of HHQ was measured by High Performance Liquid Chromatography (HPLC). The reverse-phase C_18_ Targa column (4.6 mm × 150 mm, 5 μm) (catalog number: TS-1546-C185) was used with solvent A (10 mM ammonium acetate in water) and solvent B (10 mM ammonium acetate in methanol) at a flow rate of 0.3 mL·min^−1^. The injection volume was 20 µL and 314 nm was used as the detection wavelength. The eluent gradient was as follows: 0 min, 30% B, 0 to 3 min, 70% B; 3 to 29 min, 100% B; 29 to 36 min, 100% B; 36 to 40 min, 20% B; 40 to 42 min, 20% B. The retention time of HHQ was at 22.5 min. HHQ concentrations obtained by HPLC analysis were normalized by protein concentration.

### 3.3. Pyocyanin Quantification

Bacterial cultures were grown in 10 mL of King’s medium A for 24 h at 37 °C, 200 rpm. Cell-free supernatants were collected by centrifugation and filtered through the 0.22 µm Hydrophilic Cartridge Filters (Millipore, Singapore). 5 mL of cell-free supernatants and medium control were transferred to new tubes where 1 mL of chloroform were added and mixed. The layer of chloroform at bottom was transferred to new tubes after settling. Pyocyanin was extracted from chloroform using 200 µL of 0.2 M HCl by vigorous mixing. The quantity of pyocyanin was measured by absorbance at OD_520 nm_. Pyocyanin quantities were normalized against the OD_600 nm_ values of the cultures.

### 3.4. Mixed-Species Biofilm Assay

Mixed species biofilms were established by co-culturing *S. aureus* 15981/pSB2019 and *P. aeruginosa* PAO1 wild-type, Δ*rpoN*, ∆*rpoN*COM, and Δ*rpoN*/pME6032-*pqsR* mutant, respectively, as previously described [34]. *S. aureus* 15981/pSB2019 appeared green due to gfp expression whereas *P. aeruginosa* strains were stained with red fluorescent dye, SYTO62. Imaging of biofilms was done using a Zeiss LSM780 CLSM with a 63×/1.4 objective. Imaris software package (Bitplane AG, Zürich, Switzerland) was used to generate the simulated 3D images and calculation of the biovolumes of biofilms.

### 3.5. Staphylococcus aureus Inhibitory Assay

*S. aureus* overnight cultures were washed with PBS for three times and diluted to OD_600 nm_ = 0.1. 100 μL of diluted cultures were plated evenly onto LB agar plates and spread-dried. Filter paper discs were placed onto the surface of LB agar plates on top of the *S. aureus* lawn. *P. aeruginosa* PAO1 wild-type, ∆*rpoN*, ∆*rpoN*COM, and ∆*rpoN/*pME6032-*pqsR* overnight cultures were washed and diluted to OD_600 nm_ = 0.1. 20 μL of diluted *P. aeruginosa* cultures were taken and dripped onto filter paper discs. Agar plates were then incubated at 37 °C for overnight. *S. aureus* inhibitory effect was determined by the sizes of inhibiting zones.

### 3.6. P_p**qsA**_-gfp Induction Assay

PAO1/p*_pqsA-_gfp*, ∆*rpoN*/p*_pqsA-_gfp*, ∆*rpoN*COM/p*_pqsA-_gfp*, and ∆*rpoN/*pME6032-*pqsR*/p*_pqsA-_gfp* strains were cultivated overnight in LB broth in the presence of respective antibiotics. Overnight cultures of these strains were diluted in ABTGC medium to OD_600 nm_ = 0.01, where 5 µM of external PQS signaling molecule (synthesized as previously described [15]) was added to ∆*rpoN*/p*_pqsA-_gfp* cell suspension. 200 µL of cell suspensions of each strain were loaded into wells of a 96-well microtiter plate. Six replicates of each strain were applied. Optical density at 600 nm and green fluorescence (excitation at 485 nm, emission at 535 nm) of these cultures were monitored over 24 h using a Magellen Tecan^®^ Infinite 200 PRO plate reader.

### 3.7. Caenorhabditis elegans Killing Assay

*P. aeruginosa* strains were spread as a lawn and incubated on PGS agar in 6-well plate (Nunc) at 37 °C overnight. Triplicate plates were each seeded with 20 L3-stage hermaphrodite *C. elegans* strain N2 (Bristol) [21]. Plates were incubated at 25 °C for 24 h, for the animals to feed on the bacterial lawn. Dead and live animals were enumerated and the % dead over total animals was tabulated.

## Supplementary Materials

Supplementary materials can be found at http://www.mdpi.com/1422-0067/16/12/26103/s1.
