# Supplementary material for: RpoN Regulates Virulence Factors of Pseudomonas aeruginosa via Modulating the PqsR Quorum Sensing Regulator
Source: Int J Mol Sci. 2015 Nov 30;16(12):28311–9. doi: 10.3390/ijms161226103 (PMC4691050; doi:10.3390/ijms161226103)
Supplement: Supplementary file 1 [file ijms-16-26103-s001.pdf]

Supplementary Materials: RpoN Regulates Virulence Factors of *Pseudomonas aeruginosa* via Modulating the PqsR Quorum Sensing Regulator

Zhao Cai, Yang Liu, Yicai Chen, Joey Kuok Hoong Yam, Su Chuen Chew, Song Lin Chua, Ke Wang, Michael Givskov and Liang Yang

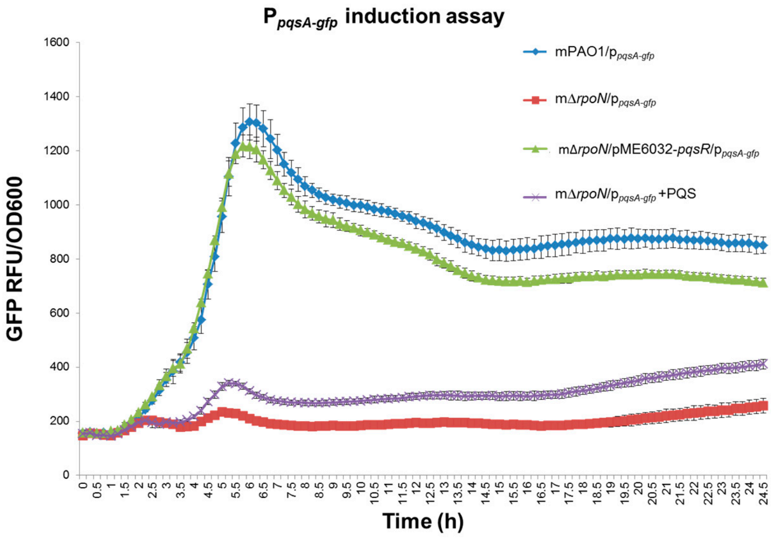

Figure S1. Induction of p*pqsA*-*gfp* transcriptional fusion in mPAO1 wild-type, mPAO1/Δ*rpoN*, mPAO1/Δ*rpoN*/pME6032-*pqsR* and mPAO1/Δ*rpoN* + PQS (2-heptyl-3-hydroxy-4(1H)-quinolone) (10 μM). Cultures were monitored for their gfp fluorescence by using a Tecan Infinite Pro2000 microplate reader. Means and standard deviations (S.D.) in relative fluorescence units (RFU) from triplicate experiments are shown.
